# Metabolome and transcriptome analyses identify the plant immunity systems that facilitate sesquiterpene and lignan biosynthesis in *Syringa pinnatifolia* Hemsl.

**DOI:** 10.1186/s12870-022-03537-5

**Published:** 2022-03-22

**Authors:** Jiaqi Gao, Tianxiao Li, Lichao Jiao, Chao Jiang, Suyile Chen, Luqi Huang, Juan Liu

**Affiliations:** 1 Institute of Traditional Chinese Medicine, Tianjin University of Traditional Chinese Medicine, 301617 Tianjin, China; 2 National Resource Center for Chinese Materia Medica, China Academy of Chinese Medical Sciences, 100700 Beijing, China; 3 Research Institute of Wood Industry, Chinese Academy of Forestry, 100091 Beijing, China; 4 Alashan Mongolian Hospital, Alashan East Banner of Alashan, 75030 Inner Mongolia, China

**Keywords:** *Syringa pinnatifolia* Hemsl., Metabolome, Transcriptome, Sesquiterpene, Lignan, Plant immunity

## Abstract

**Background:**

*Syringa pinnatifolia* Hemsl. is a shrub belonging to the Oleaceae family. The peeled woody stems and roots of *S. pinnatifolia* are used in Chinese traditional medicine. This plant has been used for centuries, and modern pharmacological research has revealed its medicinal value. However, the wild populations of *S. pinnatifolia* have been decreasing, and it has been listed as an endangered plant in China. To elucidate the molecular mechanism leading to the synthesis of the major components of *S. pinnatifolia* for its further development and sustainable use, this study compared peeled stems and twigs at the metabolic and molecular levels.

**Results:**

Peeled stems with the purple substance visible (SSP) and peeled twigs without the purple substance (TSP) were compared at different levels. Microscopic observation showed resin-like fillers in SSP and wood fiber cell walls approximately 1.0 μm thicker than those in TSP (wood fiber cell thickness approximately 2.7 μm). In addition, 104 volatile organic compounds and 870 non-volatile metabolites were detected in the non-targeted and widely-targeted metabolome analyses, respectively. Among the 76 differentially accumulated metabolites (DAMs) detected, 62 were up-accumulated in SSP. Most of these DAMs were terpenes, of which 90% were identified as sesquiterpenes in the volatile organic compound analysis. In the analysis of the non-volatile metabolites, 21 differentially accumulated lignans were identified, of which 18, including five subtypes, were accumulated in SSP. RNA sequencing revealed 4,421 upregulated differentially expressed genes (DEGs) and 5,522 downregulated DEGs in SSP compared with TSP, as well as 33,452 genes that were not differentially expressed. Analysis of the DEGs suggested that sesquiterpenes and lignans were mostly biosynthesized via the mevalonate and phenylpropanoid pathways, respectively. Additionally, in SSP, the enriched Gene Ontology terms included response to biotic stimulus and defense response, while the enriched Kyoto Encyclopedia of Genes and Genomes pathways included plant–pathogen interaction and many other pathways related to plant immunity.

**Conclusions:**

This study provides metabolome and transcriptome information for *S. pinnatifolia*, suggesting that biotic stimuli, including pathogens, are potential and valuable approaches to promoting the biosynthesis of the metabolites linked to the medicinal properties of this plant.

**Supplementary Information:**

The online version contains supplementary material available at 10.1186/s12870-022-03537-5.

## Background

**Fig. 1:**
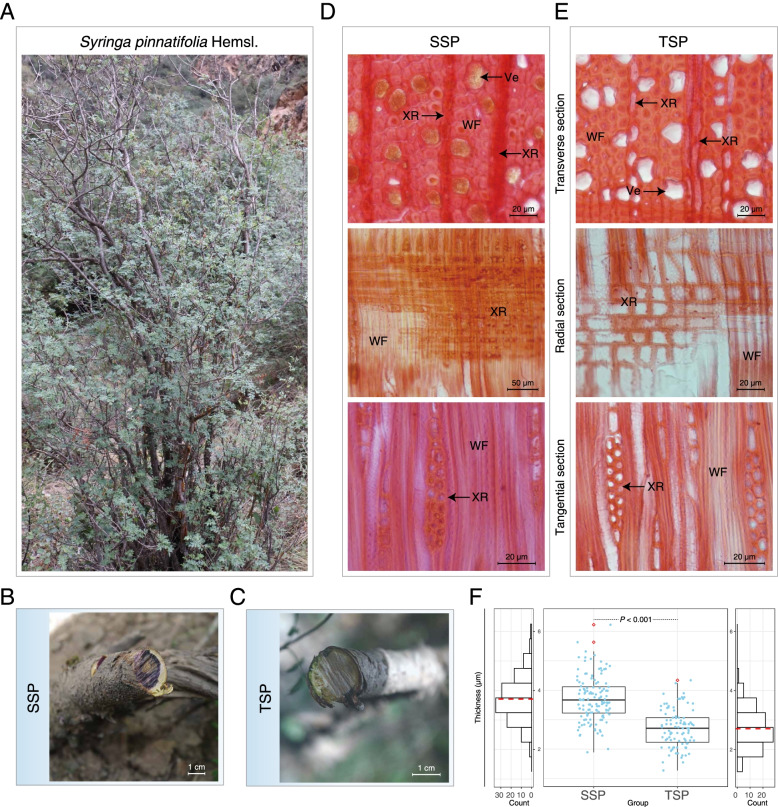
Phenotypical and morphological analyses of the plant materials. **A** Whole *S. pinnatifolia*. **B** Thick stems of *S. pinnatifolia* with the purple substance visible (SSP). **C** Thin twigs of *S. pinnatifolia* without the purple substance (TSP). **D** Transverse (top), radial (middle), and tangential (bottom) sections of SSP. **E** Transverse (top), radial (middle), and tangential (bottom) sections of TSP. **F** The distribution of wood fiber cell wall thickness in SSP and TSP. The rhombi in boxplots indicate outliers, and the red dotted lines indicate the average values. (Ve, vessel; WF, wood fiber cell; XR, xylem ray)

*Syringa* (Lilac), a popular genus of ornamental flowering woody plants, is widely cultivated in Europe, Asia, and America [1]. *Syringa pinnatifolia* Hemsl., a species endemic to China, is not only a garden plant but also a traditional medicinal herb used in the northwest part of China, especially in Inner Mongolia [2] (Fig. 1A). After pruning the plant to discard the thin twigs and lateral roots, the peeled woody parts of mature *S. pinnatifolia* are acquired, including the thick stems and roots with the purple substance visible on the inside, which are called “Shanchenxiang” (SCX) in Chinese. This name is similar to the Chinese name for agarwood (Chenxiang) [2, 3] and this material can indeed be used as a substitute for agarwood in some prescriptions of traditional Mongolian medicine [2]. Some studies have explored the components and pharmacological effects of *S. pinnatifolia* and have revealed that terpenes, especially sesquiterpenes and lignans, are the main types of chemicals in this plant [4] that cause a series of pharmacological effects. For example, the sesquiterpenes extracted from *S. pinnatifolia*, including (+)-alashanoid E, (±)-alashanoid F, and zerumbone, can exert cardioprotective effects [5], while guai-9-en-4*β*-ol and 14,15-dinorguai-1,11-dien-9,10-dione have antibacterial properties against *Bacillus coagulans* and *Escherichia coli* [6]. Lignans like noralashinol B can induce apoptosis of HepG2 cells [7], and sanshodiol and alashinols A and D have anti-inflammatory effects [8]. However, the mechanism leading to these properties of SCX remains undisclosed.

Terpenes are the largest class of small-molecule natural chemicals [9], and they can be biosynthesized in the mevalonate (MVA) and 2-C-methyl-D-erythritol 4-phosphate (MEP) pathways [10]. According to previous studies, 3-hydroxy-3-methylglutaryl-CoA reductase (HMGR), the enzyme that synthesizes MVA, is the rate-limiting enzyme in the MVA pathway [11]. One of the rate-limiting enzymes in the MEP pathway is 1-deoxy-d-xylulose 5-phosphate synthase (DXS) [12], which produces 1-deoxy-D-xylulose 5-phosphate (DXP) via the condensation of pyruvate and glyceraldehyde-3-phosphate (GA-3P) [13, 14]. The gene coding for DXP reductoisomerase (*DXR*) also has a rate-limiting function in the MEP pathway [15], in addition to influencing the development of *Arabidopsis* [16]. Sesquiterpene synthases play an important role in the synthesis of sesquiterpenes using farnesyl diphosphate (FPP) as the substrate [10], and their products vary depending on the characteristics of the different sesquiterpene synthases [17]. Lignans are phenylpropanoid dimers that are widely distributed in plants [18]. They are synthesized through the phenylpropanoid pathway [19] in which phenylalanine ammonia lyase (PAL) and dirigent protein (DP) participate [18]. PAL is a rate-limiting enzyme in the phenylpropanoid pathway and serves as a link between primary and secondary metabolism [20]. As an asymmetric inducer, DP can induce the production of pinoresinol, an important intermediate in lignan biosynthesis [21]. Although the biosynthesis of sesquiterpenes and lignans has been extensively studied, the molecular mechanisms responsible for the biosynthesis and regulation of sesquiterpenes and lignans in *S. pinnatifolia* remain unclear.

The plant immunity system produces chemicals for defense and resistance [22], and sometimes immune-related genes can be transferred to the progeny to facilitate their adaptation to the environment [23]. The defensive chemicals produced and stored constitutively in plant tissues are called phytoanticipins [24], whereas the compounds formed *de novo* for defense against pathogen invasion are defined as phytoalexins [25]. The regulation of the plant immunity system has been engineered to facilitate the production of phytoalexins [26]. During the production of downstream metabolites, signaling processes, including the mitogen-activated protein kinase (MAPK) cascade [27] and reactive oxygen species (ROS) production [28], are activated upstream. Transcription factors, such as those from the WRKY, AP2/ERF, and NAC families, which are activated by phosphorylation [29–31], can facilitate the expression of synthase genes to promote the biosynthesis of secondary metabolites, including sesquiterpenes [32]. For instance, a study on cotton showed that a fungal elicitor preparation could promote the expression of *WRKY1* in this plant to increase the upregulation of *CAD1-A* encoding (+)-*δ*-cadinene synthase and facilitate the production of sesquiterpenes [33]. Research on capsidiol, which is a sesquiterpenoid phytoalexin, showed that this compound is highly accumulated in defense against *Alternaria alternata* through the regulation of an ethylene response factor 2 (ERF2)-like transcription factor [34]. However, whether the plant immunity system could function in the production of terpenes and lignans in *S. pinnatifolia* has not yet been reported.

Resources of *S. pinnatifolia* are limited in China, and the plant has been on the list of Chinese rare and endangered plants since the 1980s [35]. Based on its medicinal value and rarity, it is necessary to clarify the mechanism of SCX production to guide its use and cultivation. Because root logging would kill the plant and accelerate the reduction of wild populations, stems and twigs have become the only available woody parts in recent years. In the present study, we collected as contradistinctive samples, peeled stems of *S. pinnatifolia* with the purple substance visible (SSP), in keeping with the requirement for medicinal use, and peeled twigs of *S. pinnatifolia* without the purple substance (TSP), which do not meet the medicinal requirement [2]. Microscopic observations showed significant histological differences between the two groups of samples. In the analyses of volatile and non-volatile metabolomes, there were significant differences in the major active components of SSP and TSP, including sesquiterpenes and lignans. Finally, we analyzed the expression pattern of genes related to the biosynthesis pathways of sesquiterpenes and lignans, and proposed a hypothesis about the immunity system of *S. pinnatifolia* based on transcriptome data. The results presented here create a foundation for research on improving the quality and utilization of this medicinal plant.

## Results

### Phenotypes and microscopic observations of SSP and TSP

The most significant feature traditionally used to determine the quality of medicinal *S. pinnatifolia* is the presence of a dark-purple substance distributed within its stems (Fig. 1B, C). In addition, Safranin O staining revealed other characteristics differentiating SSP and TSP (Fig. 1D and E). In SSP, some xylem rays and vessels were filled with yellow resin-like substances (Fig. 1D), which were not observed in TSP (Fig. 1E). Furthermore, in both SSP and TSP, the structures, including wood fiber cells, xylem rays, and vessels, were lignified. Based on the measurement of the microscopic structures in transverse sections, the average thickness of wood fiber cell walls was 3.7 μm in SSP and 2.7 μm in TSP. A P-value of less than 0.001 indicated that this difference in average thickness was significant (Fig. 1F).

### Analysis of volatile organic compounds related to sesquiterpenes in *S. pinnatifolia*

To profile the metabolic differences in volatile organic compounds between SSP and TSP, headspace-solid-phase microextraction-gas chromatography-mass spectrometry (HS-SPME-GC-MS) was used for non-targeted metabolome analysis. Across all samples, 104 volatile metabolites were detected using the electron impact (EI) ionization mode (Table S1). The overlapping total ions current (TIC) of the quality control (QC) mixtures (Fig S1), as well as the results of the principal component analysis (PCA) and orthogonal partial least squares-discriminant analysis (OPLS-DA) (Fig S2), confirmed that the quality of the data was good.

**Fig. 2:**
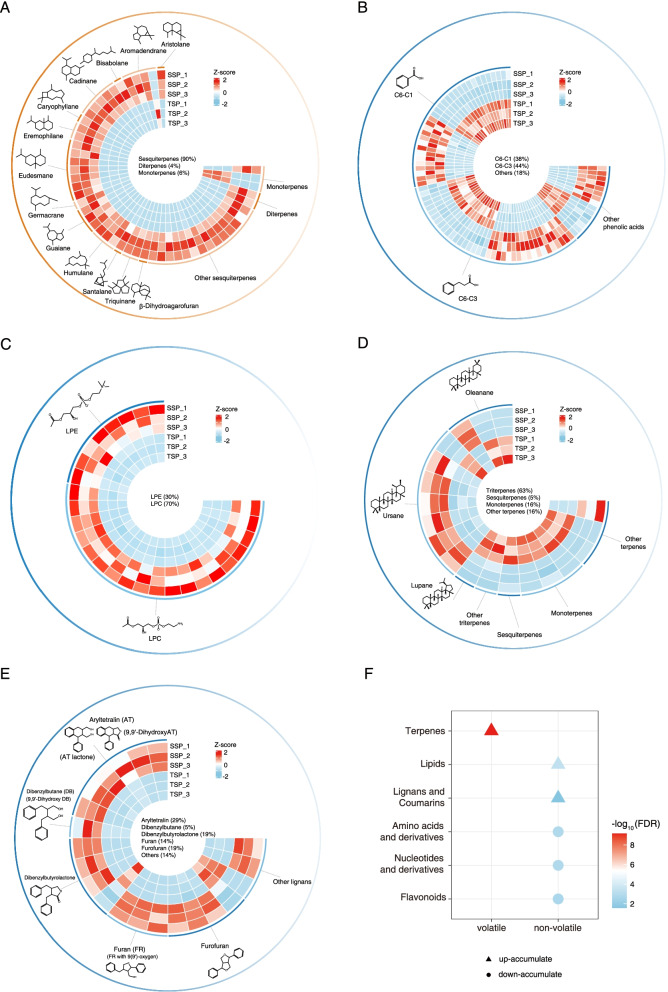
Distribution of differentially accumulated metabolites (DAMs) in the non-targeted and widely targeted metabolomes. **A** Differentially accumulated terpenes in non-targeted metabolome. **B** Differentially accumulated phenolic acids in widely targeted metabolome. **C** Differentially accumulated lysophospholipids, including lysophosphatidylcholine (LPC) and lysophosphatidylethanolamine (LPE), in widely targeted metabolome. **D** Differentially accumulated terpenes in widely targeted metabolome. **E** Differentially accumulated lignans in widely targeted metabolome. **F** Hypergeometric distribution of the main classes of DAMs. The darker red color indicates a larger value of -log_10_(FDR) and higher significance of the terms, whereas the darker blue color indicates the opposite. The other gradient color bar indicates higher up-accumulation (darker red) or higher down-accumulation (darker blue)

Of the 104 volatile metabolites detected, 55 were terpenes (Fig S3 and S4). Furthermore, nine aldehydes, alkanes, and ketones were detected, which was less than the number of terpenes (Fig S4). There were 76 differentially accumulated metabolites (DAMs) between SSP and TSP, of which 62 were found in SSP (Fig S3). Most of the DAMs were terpenes that accumulated more in SSP than in TSP, with a great proportion of up-accumulated DAMs (Figs. 2A and S3). As shown in Fig. 2A, 90% of the terpenes were sesquiterpenes belonging to 13 structural subtypes, including aristolane, aromadendrane, bisabolane, cadinane, caryophyllane, eremophilane, eudesmane, germacrane, guaiane, humulane, santalane, triquinane, *β*-dihydroagarofuran; a few diterpenes and monoterpenes were also found. Statistically, the two most abundant types of sesquiterpenes were eudesmanes and cadinanes, with seven and five metabolites, respectively. According to the distribution of the log2 fold-change (log_2_FC) values, germacrone, belonging to the germacrane type, was the metabolite with the largest value (27.274). However, isolongifolene, 4,5-dehydro- had the highest variable importance in projection (VIP) value (1.089) and the lowest false discovery rate (FDR) and P-value compared with those of other up-accumulated sesquiterpenes in SSP (Table S2), thereby revealing the potential effect of the two sesquiterpenes to differentiate SSP and TSP. According to a previous report [36], zerumbone is the humulane-type sesquiterpene with the highest content in volatile oils from *S. pinnatifolia*. Zerumbone was also detected in this experiment, with greater accumulation in SSP than in TSP (Fig. 2A; Table S2).

Only three terpenes, including an aromadendrane-type sesquiterpene and two monoterpenes, were down-accumulated in SSP (Fig. 2A). The down-accumulated aromadendrane-type sesquiterpene was ledane (XMW0195), which has a structure similar to that of ledol (XMW0027), another aromadendrane-type sesquiterpenoid that accumulated in SSP. The difference between the two is that ledol has an extra hydroxyl group (Fig. 2A; Table S2).

### Analysis of non-volatile organic compounds related to lignans in *S. pinnatifolia*

Because some compounds, such as triterpenes and lignans, can hardly be detected using the same method as that of volatile organic compounds, ultra-high-performance liquid chromatography quadrupole trap tandem mass spectrometry (UHPLC-QTRAP-MS/MS) was applied in a widely targeted metabolome analysis. There were 870 metabolites; 402 of these metabolites were detected and identified in the positive ionization mode, 468 in the negative ionization mode, and 765 identified without isomers (Table S3). Based on the results of the overlapping TIC of the QC samples (Fig S5) and cluster analyses, including PCA and OPLS-DA (Fig S6), the quality of the widely targeted metabolome data was considered reliable.

Phenolic acids and lipids were the two most abundant classifications of DAMs (Fig S7). The number of up- and down-accumulated phenolic acids was 40 and 44, respectively (Fig. 2B). Meanwhile, the main types, including C6-C1 and C6-C3, accounted for 38% and 44%, respectively, of the differentially accumulated phenolic acids. However, there was no statistical significance between up- and down-regulated phenolic acids according to the hypergeometric distribution (Fig. 2F). Additionally, 40% of the differentially accumulated lipids could be classified as lysophospholipids, and all detected lysophosphatidylcholine (LPC) and lysophosphatidylethanolamine (LPE) belonging to lysophospholipids showed higher contents in SSP than in TSP, with an average log_2_FC of 3.69 (Fig. 2C).

As important compounds in *S. pinnatifolia*, most of the terpenes were identified as triterpenes without volatility using the widely targeted metabolome and a differential analysis compared with those of the non-targeted metabolome (Fig. 2D). Ursane-type triterpenes accounted for the largest number of triterpenes detected, most of which accumulated in SSP. Thus, it was clear that volatile terpenes in the non-targeted metabolome were more abundant (Fig. 2A, D). Lignans are also an important class of chemicals with pharmacological effects in *S. pinnatifolia*, and most of them accumulated in SSP (Fig. 2E). The lignans in the DAMs contained a variety of basic skeletons, such as aryltetralin, dibenzylbutane, dibenzylbutyrolactone, furan, and furofuran.

Moreover, the contents of flavonoids, nucleotides, amino acids, and their derivatives were downregulated in SSP with statistical significance based on the hypergeometric distribution (Fig. 2F). Overall, except for the volatile terpenes, which were the most significant class of compounds accumulated in SSP, the significantly downregulated chemical classes were all non-volatile substances. Nevertheless, lipids, lignans, and coumarins were other groups of non-volatile organic compounds that also accumulated in SSP (Fig. 2F).

### Profiles of RNA sequencing and transcriptome differences between SSP and TSP

Through RNA sequencing technology, clean reads with at least 6 G-bases across all *S. pinnatifolia* samples were acquired after screening, and their error rates were no more than 3% (Table S4). Moreover, the values of Q20 and Q30 of the sequencing data all exceeded 92%, with GC contents between 40% and 50% (Table S4). These indices verified the quality of the sequencing data. Subsequently, the clean reads were assembled into transcripts with the N50 of 1,532 nucleotides; other detailed parameters are summarized in Table S5. In addition, the samples were grouped into two clusters according to the PCA and OPLS-DA plots (Fig S8). Gene functional annotation was performed using the NCBI non-redundant protein sequences (NR), NCBI nucleotide sequences (NT), Pfam, SwissProt, Kyoto Encyclopedia of Genes and Genomes (KEGG), Gene Ontology (GO), and Clusters of orthologous groups for eukaryotic complete genomes (KOG) databases. A total of 99,195 genes were annotated in at least one of these databases (Fig S9).

Differentially expressed genes (DEGs) were screened under the negative binomial generalized log-linear model. Genes whose expression value in a group was larger than that in the other group by FC > 2 and FDR < 0.05 were considered as DEGs. Setting TSP as the baseline, 4,421 DEGs were upregulated in SSP, while the number of downregulated DEGs was 5,522. In addition, 33,452 genes without significant differences in expression were identified (Fig S10).

### Overrepresentation analyses of DEGs and verification using quantitative reverse transcription-PCR (qRT-PCR)

**Fig. 3:**
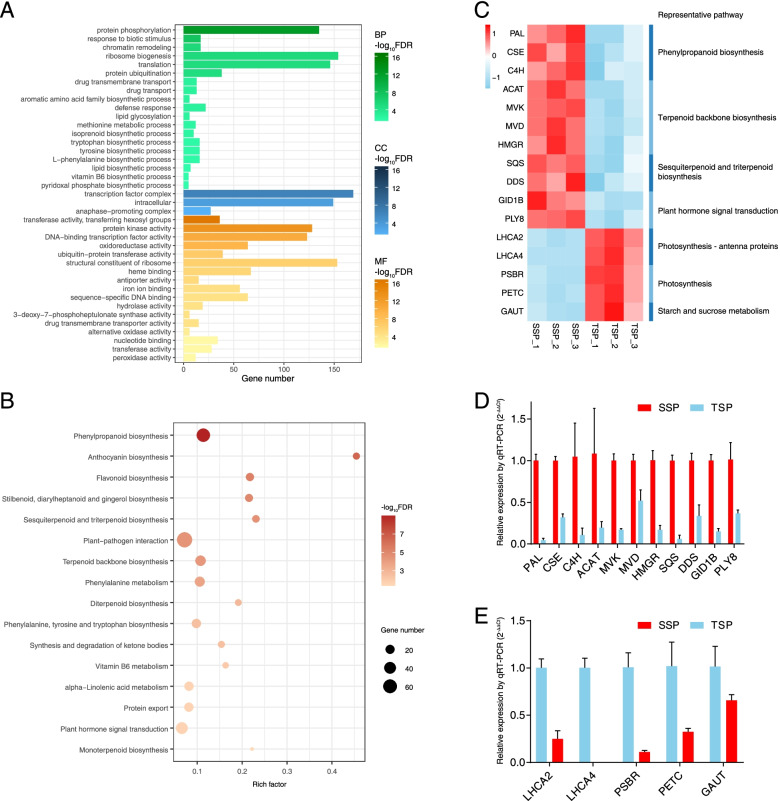
Enrichment analyses of DEGs. **A** GO enrichment analysis of upregulated DEGs in SSP. **B** KEGG enrichment analysis of upregulated DEGs in SSP. **C** Heatmap of the FPKM value of selected DEGs from the transcriptome. **D** & **E** Results of the qRT-PCR analyses of the up- and down regulated DEGs. The standard deviation (SD) is presented as the error bar. The deeper colors in gradient color bars or bubbles indicate larger values of -log_2_(FDR), pointing to the higher significance of each term or pathway

To profile the DEGs in *S. pinnatifolia*, overrepresentation analyses, including GO and KEGG enrichment analyses, were performed (Fig. 3A, B). The most significant biological process (BP) term that was upregulated in SSP was “protein phosphorylation,” indicating that this process may be more widespread in this group than in TSP. The second most important BP term was “response to biotic stimulus,” and it may be a clue for environmental stress in SSP. In addition, the significant cellular component (CC) terms presented in Fig. 3A show that the molecular activities should be more active in the transcription factor complex and intracellular areas of SSP than in those of TSP. As an example, the most significant upregulated BP term in TSP was “photosynthesis,” showing a difference between SSP and TSP (Fig S11A).

There were more KEGG pathways enriched in upregulated DEGs in SSP than in those in TSP (Fig. 3B and S11B). Similar to GO enrichment, pathways involved in secondary metabolite biosynthesis were more active in SSP than in TSP (Fig. 3B). In particular, many up-regulated pathways in SSP were related to the biosynthesis of terpenes, such as “terpenoid backbone biosynthesis,” “monoterpenoid biosynthesis,” “diterpenoid biosynthesis,” and “sesquiterpenoid and triterpenoid biosynthesis,” as well as there were some pathways involved in lignan biosynthesis, such as “phenylpropanoid biosynthesis” and “phenylalanine metabolism”, of which “phenylpropanoid biosynthesis” was the most significant (Fig. 3B). In addition, the “plant-pathogen interaction” pathway was significantly enriched (Fig. 3B), and it could be related to stress according to the GO enrichment (Fig. 3A). Apart from the pathways upregulated in SSP, the pathways upregulated in TSP showed that the most significant pathway was “photosynthesis” (Fig S11B), which was similar to the GO enrichment results (Fig. 3A). Additionally, the upregulation of the pathway of starch and sucrose metabolism in TSP may be related to the activity of the photosystem in this group.

To confirm the reliability of the overrepresentation analysis and transcriptome data, qRT-PCR was conducted on 16 selected DEGs. The selected DEGs denoted seven representative pathways that were significantly up- or downregulated (Fig. 3C), including the “phenylpropanoid biosynthesis” and “photosynthesis” pathways, which were the most significant in SSP and TSP, respectively (Fig. 3B and S11B). Based on the transcriptome data, the genes upregulated in SSP were approximately 7.6 times more highly expressed in SSP than in TSP, as indicated by the qRT-PCR results (Fig. 3D). Additionally, the genes upregulated in SSP showed approximately 4.4 times lower expression in TSP, based on the results of qRT-PCR, except for *LHCA4*, whose expression was not detected in TSP samples (Fig. 3E). The above-mentioned results proved the reliability of the transcriptome data.

### Biosynthetic pathways of sesquiterpenes and lignans

**Fig. 4:**
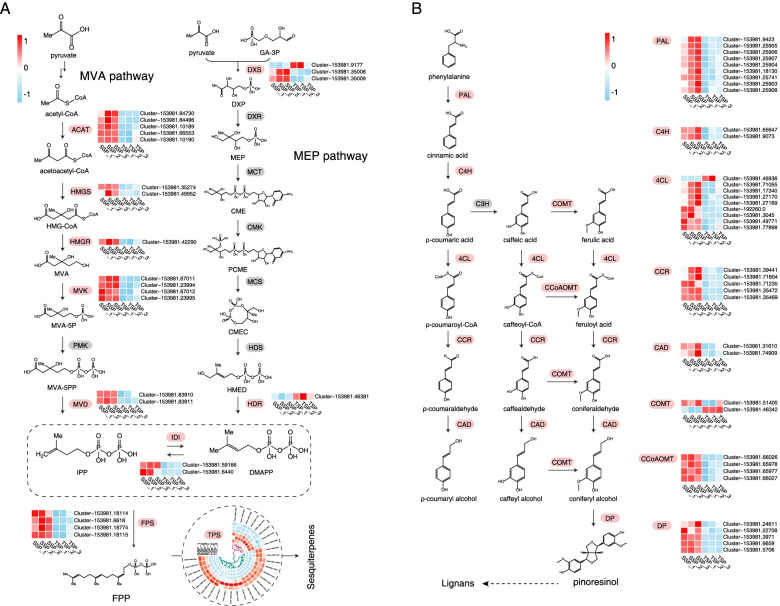
Biosynthetic pathways of the major compounds. **A** Biosynthetic pathway of sesquiterpenes. **B** Biosynthetic pathway of lignans. Pink color blocks indicate the presence of DEGs, whereas gray color blocks indicate that no DEGs are present. The gradient color bars demonstrate the z-score standardized from the FPKM value to present gene expression

According to a previous study [4] and our metabolome data (Fig. 2), terpenes, particularly sesquiterpenes, are important factors affecting the medicinal value of *S. pinnatifolia*. To reveal the characteristics of the molecular biosynthesis pathway of sesquiterpenes, the DEGs associated with terpenoid backbone biosynthesis and downstream pathways were analyzed (Fig. 4A). We found that most DEGs in the MVA pathway, including *HMGR* with its key rate-limiting ability, showed approximately six times higher expression in SSP than in TSP based on the qRT-PCR data (Fig. 3D). This is despite the non-significant difference in the expression of *PMK* in this pathway (Fig. 4A). This evidence suggested that the MVA pathway is dominant in the biosynthesis of sesquiterpenes in SSP. Additionally, 29 DEGs were annotated as potential sesquiterpene synthase genes, of which 21 were upregulated in SSP (Table S6). The most significant potential sesquiterpene synthase genes encoded premnaspirodiene oxygenase and vetispiradiene synthase. These enzymes are involved in the biosynthesis of spirovetivane-type sesquiterpenes, which were not detected in our metabolome analyses (Fig. 2A, D).

Regarding the biosynthetic pathway of lignans in *S. pinnatifolia*, almost all DEGs were upregulated in SSP, except for *4CL* and *COMT* (Fig. 4B). In particular, *PAL*, which encodes the enzyme that catalyzes the first and rate-limiting step in the phenylpropanoid pathway, was more highly expressed (by approximately 22 times) in SSP than in TSP according to the qRT-PCR results (Fig. 3D). This demonstrated that the pathway for lignan biosynthesis was very active in SSP. In fact, the upstream pathway of lignan biosynthesis was similar to that of lignin biosynthesis [37], which may explain the hardness of the woody stem of mature *S. pinnatifolia* plants.

### Gene expression of the DEGs involved in plant immunity

**Fig. 5:**
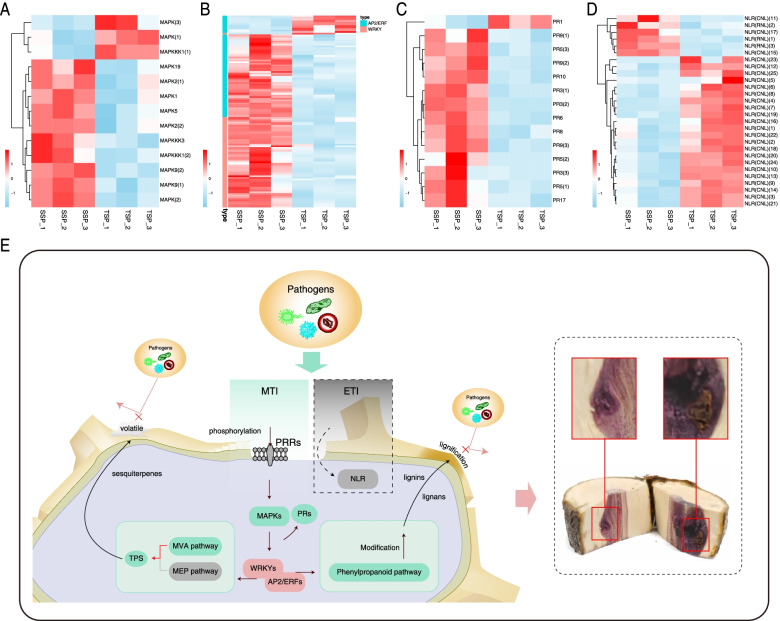
Heatmaps of the DEGs involved in plant immunity and hypothesized mechanism of the production of high-quality medicinal *S. pinnatifolia*. **A** Gene expression of differentially expressed *MAPKs.* **B** Gene expression of differentially expressed *AP2/ERFs* and *WRKYs*, which were the top two differentially expressed genes encoding transcription factors. **C** Gene expression of differentially expressed pathogenesis-related genes. **D** Expression of differentially expressed NLR family genes. **E** Hypothesized mechanism to produce high-quality medicinal *S. pinnatifolia*. The bars with different colors next to the heatmap indicate different transcription factor families in (**B**). The substances and gray blocks surrounded by dotted lines indicate an auxiliary pathway, whereas the solid lines demonstrate the major routes in (**E**)

The plant-pathogen interaction pathway was significant in both SSP and TSP (Fig. 3B and S11B), indicating the importance of biotic stress. As an important part of the signaling process under biotic stress [27], there were 13 DEGs encoding MAPKs, 10 of which were upregulated in SSP (Fig. 5A). Based on the DEG analysis using edgeR, *MAPK9(2)* was the DEG with the highest log_2_FC value (6.82) and the lowest FDR value among differentially expressed *MAPKs* (Table S7). In addition, all downregulated *MAPKs* in SSP were ranked among the bottom three owing to their FDR values, with a lower significance than that of the upregulated *MAPKs*. Most of the AP2/ERF and WRKY transcription factors that are important in the MAPK cascade and its downstream regulation [29, 38] were upregulated in SSP (Fig. 5B). In particular, the AP2/ERF and WRKY transcription factors were the top two differentially expressed transcription factors, and only one *WRKY* was downregulated in SSP (Fig. 5B and S12), implying that they are important in SSP.

Concerning the DEGs encoding pathogenesis-related (PR) proteins, except for one gene belonging to the PR1 subfamily, 13 *PRs* were upregulated in SSP (Fig. 5C). Among the differentially expressed *PRs*, *PR3(2)* was listed first, presenting the highest log_2_FC value (10.11) as well as the lowest FDR (Table S8). Furthermore, the only downregulated gene had the second highest FDR value compared with those of the other *PRs*. In addition, of the 28 nucleotide-binding leucine-rich repeat (NB-LRR or NLR) genes, which are typical plant resistance (R) genes, 22 were downregulated in SSP (Fig. 5D) showing a different pattern from that of the *PRs*.

## Discussion

According to recent studies, many new sesquiterpenes and lignans have been identified in *S. pinnatifolia* [8, 39, 40]. Therefore, this woody plant is a valuable resource for discovering new compounds, especially sesquiterpenes and lignans, which may be regarded as potentially important chemicals in the development of medicines because of their abundance in *S. pinnatifolia* [4]. To determine the profile of the metabolites, especially sesquiterpenes and lignans, in the production of high-quality SCX, a metabolome analysis was performed (Fig. 2). Sesquiterpenes and lignans were the major volatile and non-volatile compounds because they accumulated in greater concentrations in SSP than in TSP (Fig. 2A, E, F). The types of sesquiterpenes varied, but there was no dominant type in terms of quantity in significant DAMs. Although zerumbone was the most abundant component in volatile oils based on a previous report [36], it was not the most significant sesquiterpene despite its differential accumulation in SSP and TSP, suggesting that some other sesquiterpenes may have more significant effects and that the sesquiterpenes in SCX may be more active as mixtures. Regarding triterpenes, belonging to terpenes, the ursane-type was the main up-accumulated DAM (Fig. 2D), suggesting another direction to study non-volatile terpenes. Thus, the separation and identification of potential chemicals are still valuable tools in improving modern medicine.

Apart from sesquiterpenes and lignans, lipids were also significantly up-accumulated in SSP (Fig. 2F). The yellow resin-like substance in SSP (Fig. 1) may be a mixture of these metabolites, especially of the lysophospholipids containing LPC and LPE, of which the LPE were more up-accumulated in SSP than in TSP (Fig. 2C). According to previous studies, the content of LPC is often low in plants, but it increases under some special conditions, such as freezing stress [41]. Furthermore, LPC can activate some signal transductions to influence stress responses [42, 43]. Therefore, lipids, especially LPC, should be active in the production of high-quality *S. pinnatifolia*, and this process may be related to environmental stress because this plant grows at high altitudes [44].

The proposed biosynthesis processes of sesquiterpenes and lignans using RNA sequencing are illustrated in Fig. 4. In the biosynthesis of terpenes, the MVA and MEP pathways are two important upstream pathways that occur in the cytoplasm and plastids, respectively [45]. For sesquiterpenes, it was clear that the main upstream pathway in SSP was the MVA pathway, which is a common pathway in the biosynthesis of sesquiterpenes, sterols, triterpenes, and their derivatives in plants [46]. Generally, monoterpenes, diterpenes, and tetraterpenes are synthesized via the MEP pathway [46], and this may explain why most of the terpenes were sesquiterpenes, followed by some triterpenes. Regarding their physiological effects on plants, sesquiterpenes are effective in the defense against biotic and abiotic stresses, thereby protecting the plants [47]. For example, (*E*)-*β*-farnesene can be produced in *Arabidopsis thaliana* to repel the green peach aphid [48], and nematodes are attracted by the (*E*)-*β*-caryophyllene emitted from maize to attack insect larvae, thereby protecting the roots [49]. By taking advantage of these characteristics, many studies have been conducted to increase the yield of sesquiterpenes. For instance, researchers have adopted various methods, including mechanical wounding, biological inoculation, and chemical stimulation, to facilitate the formation of these chemicals in agarwood, which has sesquiterpenes as major effective components [50].

Lignan is another important component of *S. pinnatifolia*, although research has only identified the biosynthesis pathways of a few lignans [37]. The upstream pathways of lignan and lignin biosynthesis are similar according to previous reports [37], and both compounds are important in defense responses. Lignin is a major component of the cell wall of vascular plants and can facilitate lignification to allow plants to defend against external attacks [51]. Lignans also play a role in defense; for example, they can act as antifeedants and inhibit insect feeding invasions [52]. Some researchers have claimed that the induction of cinnamyl alcohol dehydrogenase (CAD), with catalytic capacity in the biosynthesis of lignin and lignan, could be treated as a universal marker of plant defense [53]. The two differentially expressed *CADs*, as well as the *PALs*, which are also well-recognized genes involved in pathogen defense [54], were all upregulated in SSP (Fig. 4B).

Protein phosphorylation is a type of post-translational modification involved in the plant immunity system in response to pathogens [55], and it was also the most significant BP term in SSP (Fig. 3A). When plants are invaded by pathogens, MAPKs are activated to trigger the MAPK cascade after the phosphorylation of pattern recognition receptors (PRRs), such as FLS2, BKA1, and their complexes in *A. thaliana* [56, 57] with the aim of plant protection [27]. Previous studies have shown that AP2/ERF and WRKY transcription factors play important roles in the connection of the pathogen-activated MAPK cascade with downstream reactions of transcription [29, 38]. For example, two identified *AP2/ERF*s could promote the expression of *ADS* and *CYP71AV1*, which are key genes in the biosynthesis of artemisinin, a well-known and important sesquiterpene used in the treatment of malaria [58]. *Ii049*, belonging to the AP2/ERF family, could bind to the coupled elements of *PAL* and *CCR* to facilitate lignan biosynthesis in *Isatis indigotica* [59]. WRKY is another transcription factor family that regulates germination; senescence; development; biotic and abiotic stresses [60]; and the biosynthesis of three major classes of metabolites, namely, phenylpropanoids, alkaloids, and terpenes [61]. Similar to the *AP2/ERFs*, a WRKY family transcription factor (*AaGSW1*) could regulate *CYP71AV1* and *AaORA* expression to increase the yield of artemisinin and dihydroartemisinic acid in *Artemisia annua* [62]. Furthermore, in the study of *I. indigotica*, *IiWRKY34* could regulate lignan biosynthesis and stress tolerance [63].

In addition, *PR* genes are a downstream indicator of the activation of plant immunity [64]. According to our results (Fig. 5A–C), most of the above-mentioned genes were upregulated in SSP. In addition, based on previous research on 16 S rDNA amplicon sequencing of *S. pinnatifolia*, wild woody stems showing the purple substance had more diverse bacterial species than samples without the purple substance [65]. These findings show that pathogen stimulus and the plant immunity system are important for the formation of SCX.

In plant immunity, there are two main mechanisms, microbial-associated molecular pattern-triggered immunity (MTI) and effector-triggered immunity (ETI). Some downstream responses of MTI, such as the MAPK cascade and the activation of *PR* genes, overlap with those of ETI [23]. In the ETI approach, *NLR* genes, which comprise the largest family of plant resistance genes, elicit ETI to resist pathogen invasion [66, 67]. However, the differentially expressed *NLRs* showed a different pattern from that of other DEGs, such as *MAPKs* or *PRs*. Therefore, ETI may be in an auxiliary position for response in SSP.

By integrating the results of this study, a hypothetical diagram for the production mechanism of high-quality SCX is provided in Fig. 5E. Additionally, we found that there was an area suspected to have been invaded and damaged by pathogens in the SSP section, and the nearby area showed a deeper purple color than did other areas (Fig. 5E). This suggested that the purple area may be a site of pathogen invasion, corresponding with the traditional method [2] used to single out the high-quality medicinal SCX. Taken together, our results allowed us to hypothesize that the process of high-quality SCX production starts from pathogen invasion, followed by MTI initiation when the PRR signaling, such as phosphorylation, and the MAPK cascade are activated to promote the expression of transcription factors from the WRKY and AP2/ERF families and some pathogenesis-related genes. Finally, the biosynthesis pathways of secondary metabolites, including volatile sesquiterpenes, non-volatile lignans, and some lignins, are upregulated to promote the production of these metabolites in the plant’s defense against pathogens. The ETI system may be an auxiliary defense mechanism, and pathogenesis-related proteins would also be effective in the defense against pathogens [64]. High-quality SCX is produced in the perennial defense process.

It should be clear that the factors influencing the development and metabolism of plants, such as light, temperature, soil, and humidity, affect the expression of *HMGR* in *S. pinnatifolia*. Our previous study showed that low temperatures increase *HMGR* expression in *S. pinnatifolia* [68]. In the present study, the interaction of metabolite characteristics and the expression of some typical genes associated with plant immunity suggested that biotic stimulus, including pathogens and bacteria as referred to in a previous study [65], would be a potential and feasible approach to accelerate the accumulation of sesquiterpenes and lignans in the cultivation of *S. pinnatifolia*.

## Conclusions

*Syringa pinnatifolia* is not only a garden plant, but it is also an endangered traditional herb in China. To the best of our knowledge, prior to this study, metabolic comparisons and transcriptome analyses of this shrub have not yet been reported. In the present study, histological comparisons revealed the characteristics of the medicinal and non-medicinal parts of *S. pinnatifolia*. Focusing on the formation mechanism of the effective metabolites in *S. pinnatifolia*, we clarified the profile of the major metabolites, including sesquiterpenes and lignans, as well as their subtypes. After RNA sequencing, the proposed biosynthesis pathways of sesquiterpenes and lignans were described. While the MVA pathway was the main approach to synthesize sesquiterpenes, lignans were produced by the phenylpropanoid pathway. Based on the information on the phytoalexins in *S. pinnatifolia* and on the expression of the genes associated with plant immunity, we hypothesized that pathogen induction may be an important factor influencing the biosynthesis of the major metabolites, including sesquiterpenes and lignans, and that it would trigger the immune system, especially MTI, as a potential way to activate a series of regulations to produce those metabolites. These findings provide new insights into the molecular mechanism of sesquiterpene and lignan biosynthesis and are useful in the further development and protection of *S. pinnatifolia*.

## Materials and methods

### Plant materials

The peeled stems and twigs of *S. pinnatifolia* (SSP and TSP) were collected from Xiazi Gully in Mount Helan Nature Reserve (N38°29′32″, E105°50′2″), at an altitude of approximately 1900 m, in Inner Mongolia, China. The average annual precipitation in the sampling area is 200–400 mm, and the majority of the precipitation occurs from July to September [69]. In addition, the average annual evaporation and temperature are approximately 2000 mm and − 0.8 ℃, respectively [70]. The thick stem samples with the purple substance visible on the inside, corresponding to the traditional medicinal standard [2], were named SSP in this study, while the thin twig samples without the purple substance were named TSP. The source shrubs were at the mature stage, and the ages of SSP and TSP were approximately 30 and 10 years, respectively, according to their annual rings. The samples were collected in July, which is the wettest period [69] but it is also the period of the year when *S. pinnatifolia* grows most vigorously. All samples were frozen in liquid nitrogen and stored in an ultra-low temperature refrigerator at -80 ℃. The collection of *S. pinnatifolia* was permitted by the fourth national survey of Chinese matiera medica resources in China, and complied with the IUCN policy statement on research involving species at risk of extinction and the convention on the trade in endangered species of wild fauna and flora, as well as the relevant guidelines and legislation in China. The identification of *S. pinnatifolia* was performed by Dr. Suyile Chen from Alashan Mongolian Hospital, and the specimen was deposited in the herbarium of National Resource Center for Chinese Materia Medica, China Academy of Chinese Medical Sciences.

### Staining and microscopic observation

A Leica CM1860 freezing microtome (Leica Microsystems Inc., Wetzlar, Germany) was used to slice stem samples into microsections. The microsections were stained with Safranin O for subsequent microscopic observation under an Olympus EX51 microscope (Olympus Corporation, Tokyo, Japan). Measurement of the structures in photomicrographs was carried out using Image Pro-Plus (Media Cybernetics, Rockville, Maryland, USA), and the significance of differences was estimated by the Student’s *t*-test using IBM SPSS Statistics 22 (IBM, Armonk, New York, USA).

### Non-targeted metabolome analysis by HS-SPME-GC-MS

Three technical repeats of the stem or twig samples were mixed as one biological repeat, and there were three biological repeats each in the SSP and TSP samples. The stem samples were ground into powder in liquid nitrogen, added to a 20 mL headspace vial (Agilent, Palo Alto, California, USA) capped by PTFE-silicone headspace septa, and then incubated at 60 ℃ for 10 min. Subsequently, a 65 μm DVB/CAR/PDMS fiber needle (Supelco, Bellefonte, Pennsylvania, USA) was exposed to the vial for 20 min at 60 ℃. After sampling, the fiber needle was inserted into the injection port of the 7890B GC apparatus (Agilent) for desorption in the splitless mode at 250 ℃ for 5 min. The identification and quantification of the metabolites were performed using the 7890B GC and 7000D MS (Agilent) equipped with a DB-5MS capillary column (30 m × 0.25 mm × 1.0 μm). The carrier gas was helium, and the velocity was 1.0 mL/min. The injector and detector temperatures were 250 ℃ and 280 ℃, respectively. The oven was heated at 40 ℃ for 5 min, and the temperature was increased at a rate of 6 ℃/min to 280 ℃ and then held for 5 min. In EI ionization mode at 70 eV, the temperature of the quadrupole mass detector was 150 ℃ and those of the ion source and transfer line were 230 ℃ and 280 ℃, respectively. In addition, the scanning m/z range of MS was 30–350. The mixture of samples was inserted as the quality control (QC) for every 10 samples. The metabolites were identified by comparing the detected spectra with the data system library (MWGC). PCA, OPLS-DA, and differential metabolite analyses were performed using the R package MetaboAnalystR [71]. DAMs were identified for metabolites if they had an FDR < 0.05, a VIP > 1, and |log_2_FC| values > 1.

### Widely targeted metabolome analysis by UHPLC-QTRAP-MS/MS

The repeats and groups analyzed in UHPLC-QTRAP-MS/MS were the same as those analyzed in HS-SPME-GC-MS. After the grinding process, 100 mg of the resulting powder was dissolved in 1.2 mL of 70% methanol solution, vortexed for 30 s every 30 min for 3 h, and then kept in a refrigerator at 4 ℃ overnight. After centrifugation at 12,000 rpm for 10 min, the supernatant was filtered through a 0.22 μm membrane (ANPEL, Shanghai, China) before sampling. The column model in the UHPLC apparatus (Shimadzu Corporation, Kyoto, Japan) was an Agilent SB-C18 column (1.8 μm, 2.1 mm × 100 mm), and the mobile phase consisted of solvent A (pure water with 0.1% formic acid) and solvent B (acetonitrile with 0.1% formic acid). The gradient elution began with a composition of 95% A + 5% B, and it changed into 5% A + 95% B in 9 min at a linear velocity, with 1 min of holding time. Subsequently, the ratio of B decreased to 5% in 1.1 min, and it was kept for 2.9 min. The temperature of the column oven was set at 40 ℃, and the flow velocity and injection volume were 0.35 mL/min and 4 µL, respectively. Measurements were conducted using a linear ion trap (LIT) and triple quadrupole scans in the AB4500 triple quadrupole-linear ion trap mass spectrometer (Applied Biosystems, Waltham, MA, USA) equipped with an ESI Turbo Ion-Spray interface. The source temperature was set at 550 ℃ with high collision-activated dissociation. The ion spray voltages were 5500 V and 4500 V in the positive and negative ion modes, respectively. The pressure parameters of the ion source gas I (GSI), gas II (GSII), and curtain gas (CUR) were 50, 60, and 25 psi, respectively. The tuning and calibration of the system were adjusted with 100 µmol/L and 10 µmol/L polypropylene glycol solutions in the LIT and triple quadrupole modes, respectively. In the triple quadrupole scans, multiple reaction monitoring (MRM) was used for quantification, and nitrogen was used as the collision gas set to the medium. After optimization of the declustering potential and entrance potential, MRM transitions were monitored for each period based on the metabolites eluted within this period. The quality control, processing, and analysis of the data were conducted in the same manner as in HS-SPME-GC-MS.

### RNA extraction and transcriptome sequencing

Three biological replicates of SSP and TSP were used for RNA extraction in preparation for sequencing. After grinding the samples in liquid nitrogen, 200 mg of powder was used for RNA extraction with the TRIzol™ reagent (Thermo Fisher Scientific, Waltham, MA, USA) following the manufacturer’s instructions. The integrity of the RNA was examined by agarose gel electrophoresis, and RNA purity was determined using a NanoDrop2000 spectrophotometer (Thermo Fisher Scientific). Thereafter, 0.5 µg of RNA per sample was reverse-transcribed into single-stranded cDNA using the PrimeScript™ RT reagent Kit with gDNA Eraser (TakaraBio, Kusatsu, Japan), according to the manufacturer’s instructions. Second-strand cDNA was synthesized using DNA polymerase, RNase, and dNTPs. After end-repair, A-tailing, and indexing ligation, AMPure XP (Beckman Coulter, Inc., Brea, CA, USA) was used for purification. For further amplification, cDNA libraries were constructed. After diluting the cDNA libraries to 1.5 ng/µL, the Agilent 2100 bioanalyzer was used to check the insert size. Finally, RNA sequencing was performed on the Illumina HiSeq platform (Illumina, San Diego, CA, USA). The data were deposited in the Genome Sequence Archive [72] of the National Genomics Data Center [73] under the accession number CRA005390, which is publicly accessible at https://ngdc.cncb.ac.cn/gsa. Transcriptome assembly was performed using the Trinity method [74].

### Bioinformatics analysis of transcriptome data

The genes were annotated based on the NR, NT, Pfam, SwissProt, KEGG, GO, and KOG databases using Blast2GO [75], Diamond [76], KAAS [77], HMMscan [78], and BLAST+ [79]. Transcription factors were predicted using iTAK [80], and gene expression was quantified by FPKM [81] using RSEM software [82]. Differential analysis of the genes was carried out using edgeR [83] with a negative binomial generalized log-linear model. DEGs were identified when the following criteria were met: log_2_FC > 1 or < -1 and FDR < 0.05. For GO and KEGG annotations, custom datasets were constructed and used for enrichment analysis by clusterProfiler [84]. Significant GO terms or KEGG pathways were selected when FDR values were < 0.05.

### qRT-PCR analysis

The qRT-PCR was conducted using the LightCycler® 480 System (Roche, Basel, Switzerland). The reaction solution was mixed with 5 µL of TB Green Premix Ex Taq II (TakaraBio), 3 µL of nuclease-free water, 1 µL of cDNA, 0.5 µL of forward primer (10 µM), and 0.5 µL of reverse primer (10 µM). The program of the qRT-PCR was set as follows: 94 ℃ for 1 min, 40 cycles of 94 ℃ for 10 s, and 60 ℃ for 34 s. Finally, at 1.0 ℃/s, the temperature was increased from 60 ℃ to 95 ℃ to obtain the melting curves. There were four biological repeats in the qRT-PCR, each with three technical repeats. Detailed information on the primers is provided in Supplementary Table S9, and *TBP* was chosen as the reference gene based on a previous study [68]. Gene expression was calculated using the 2^−ΔΔCT^ method [85].

## Supplementary Information

**Additional file 1.**

## Data Availability

The RNA-seq data have been deposited in the Genome Sequence Archive in the National Genomics Data Center under the accession number CRA005390, which is publicly accessible at https://ngdc.cncb.ac.cn/gsa.

## Abbreviations

SSP: The peeled stems of *S. pinnatifolia* with the purple substance visible
TSP: The peeled twigs of *S. pinnatifolia* without the purple substance
DAMs: Differentially accumulated metabolites
DEGs: Differentially expressed genes
SCX: Shanchenxiang
MVA: Mevalonate
MEP: 2-C-methyl-D-erythritol 4-phosphate
HMGR: 3-hydroxy-3-methylglutaryl-CoA reductase
DXS: 1-deoxy-d-xylulose 5-phosphate synthase
DXP: 1-deoxy-D-xylulose 5-phosphate
GA-3P: Glyceraldehyde-3-phosphate
DXR: DXP reductoisomerase
FPP: Farnesyl diphosphate
PAL: Phenylalanine ammonia lyase
DP: Dirigent protein
MAPK: Mitogen-activated protein kinase
ROS: Reactive oxygen species
HS-SPME-GC-MS: Headspace-solid-phase microextraction-gas chromatography-mass spectrometry
EI: Electron impact
TIC: Total ions current
QC: Quality control
PCA: Principal component analysis
OPLS-DA: Orthogonal partial least squares-discriminant analysis
VIP: Variable importance in projection
FDR: False discovery rate
UHPLC-QTRAP-MS/MS: Ultra-high-performance liquid chromatography quadrupole trap tandem mass spectrometry
LPC: Lysophosphatidylcholine
LPE: Lysophosphatidylethanolamine
NR: NCBI non-redundant protein sequences
NT: NCBI nucleotide sequences
KEGG: Kyoto Encyclopedia of Genes and Genomes
GO: Gene Ontology
KOG: Clusters of orthologous groups for eukaryotic complete genomes
FC: Fold change
qRT-PCR: Quantitative reverse transcription-PCR
BP: Biological process
CC: Cellular component
MF: Molecular function
PR: Pathogenesis-related
NLR: Nucleotide-binding leucine-rich repeat
CAD: Cinnamyl alcohol dehydrogenase
MTI: Microbial-associated molecular pattern-triggered immunity
ETI: Effector-triggered immunity
