# Supplementary material for: Metabolome and transcriptome analyses identify the plant immunity systems that facilitate sesquiterpene and lignan biosynthesis in Syringa pinnatifolia Hemsl
Source: BMC Plant Biol. 2022 Mar 22;22:132. doi: 10.1186/s12870-022-03537-5 (PMC8939180; doi:10.1186/s12870-022-03537-5)
Supplement: Supplementary file 1 — Additional file 1. [file 12870_2022_3537_MOESM1_ESM.zip › Supplementary_Figures_and_tables.pdf]

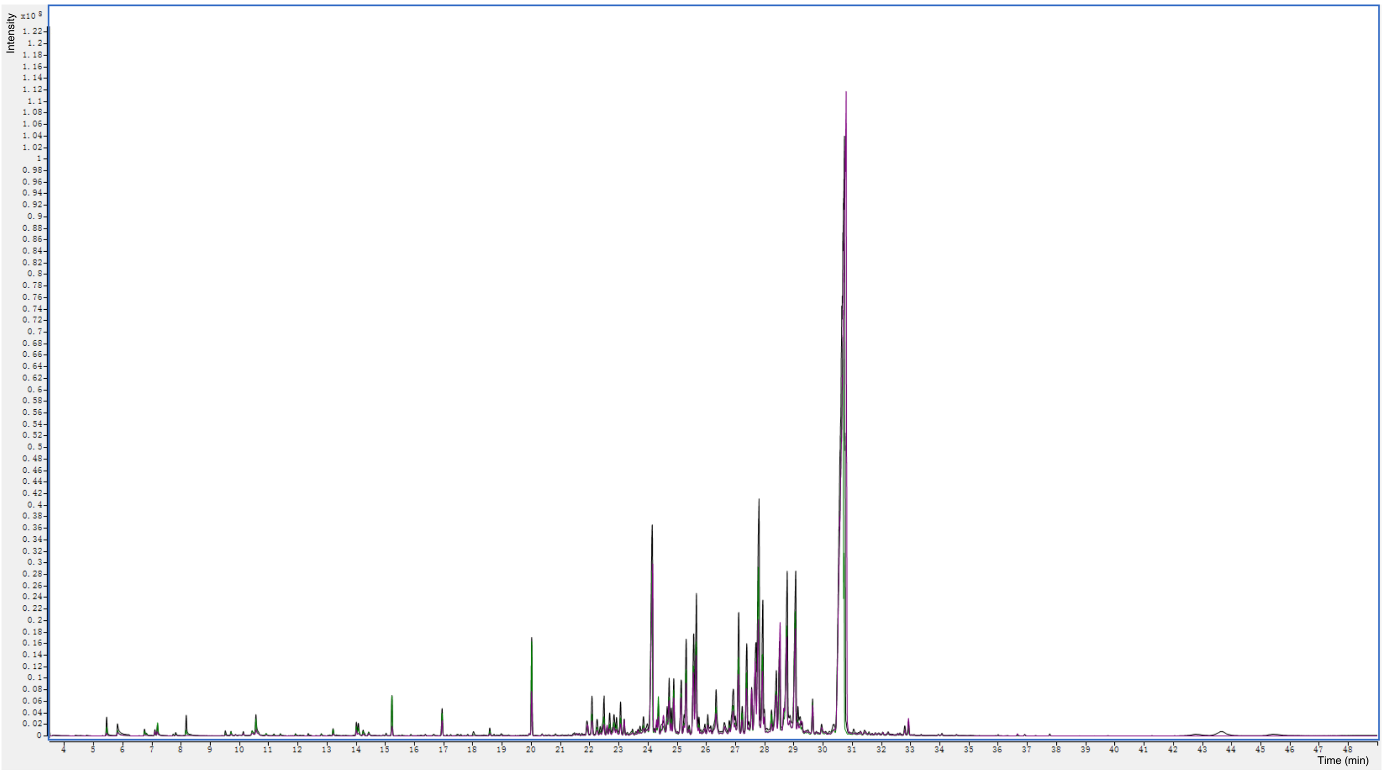

Fig. S1 Overlapping total ion current (TIC) of the quality control (QC) mixtures in the non-targeted metabolome.

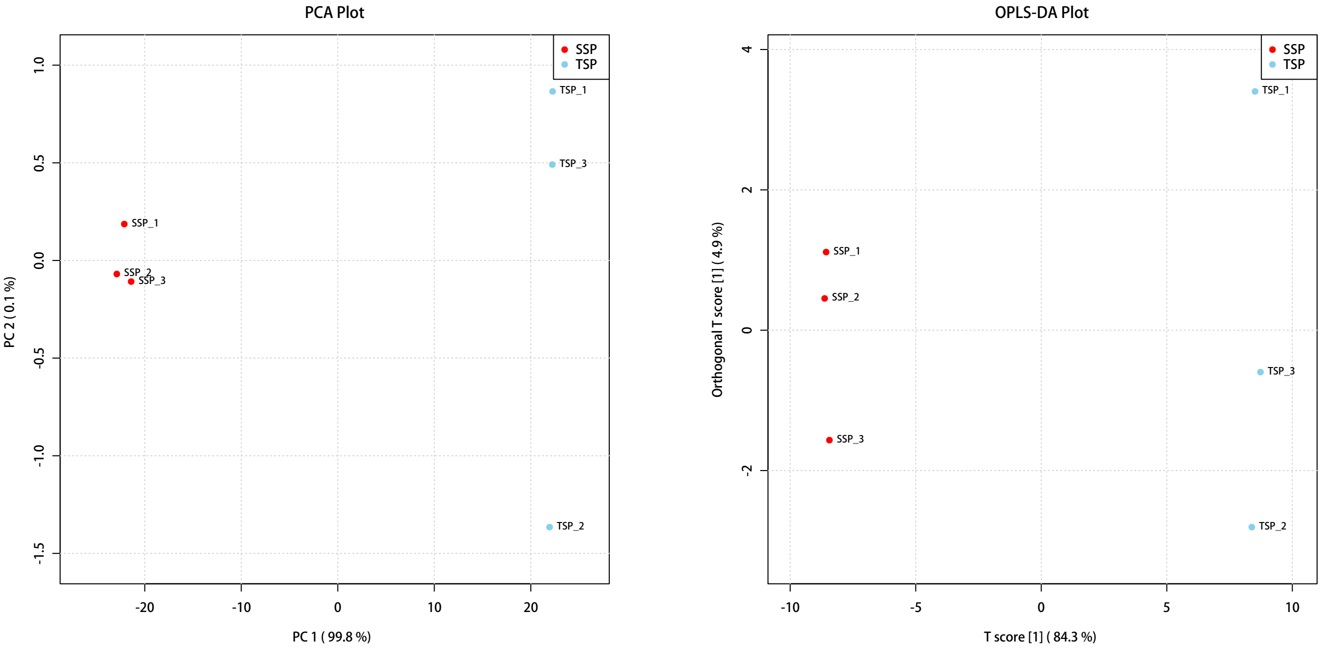

Fig. S2 Principal component analysis (PCA) and orthogonal partial least squares-discriminant analysis (OPLS-DA) plots of non-targeted metabolome data of volatile organic compounds.

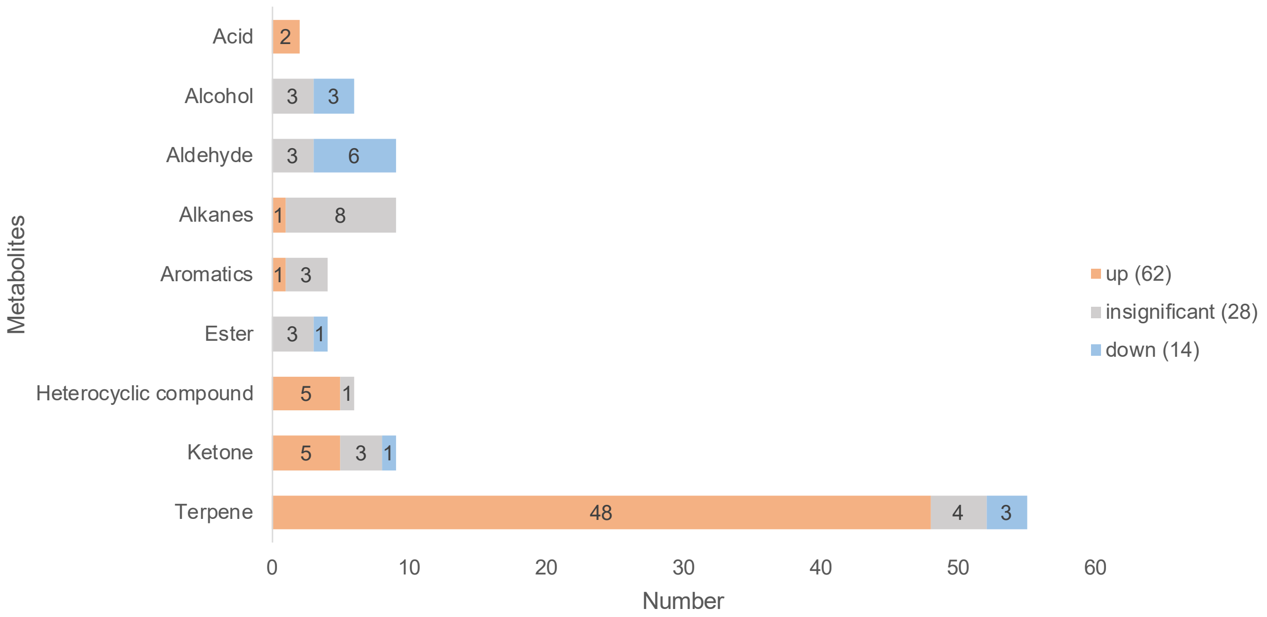

Fig. S3 Differentially accumulated metabolites in non-targeted metabolome.

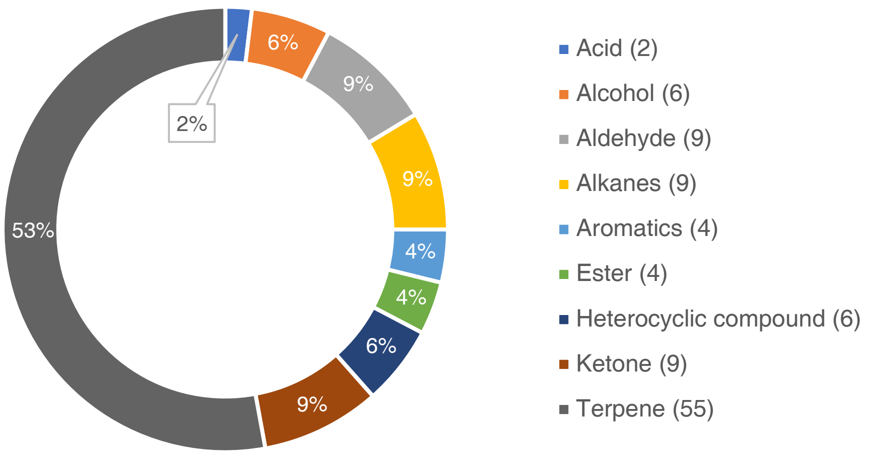

Fig. S4 Distribution of the chemical classification of the detected volatile organic compounds in the non-targeted metabolome.

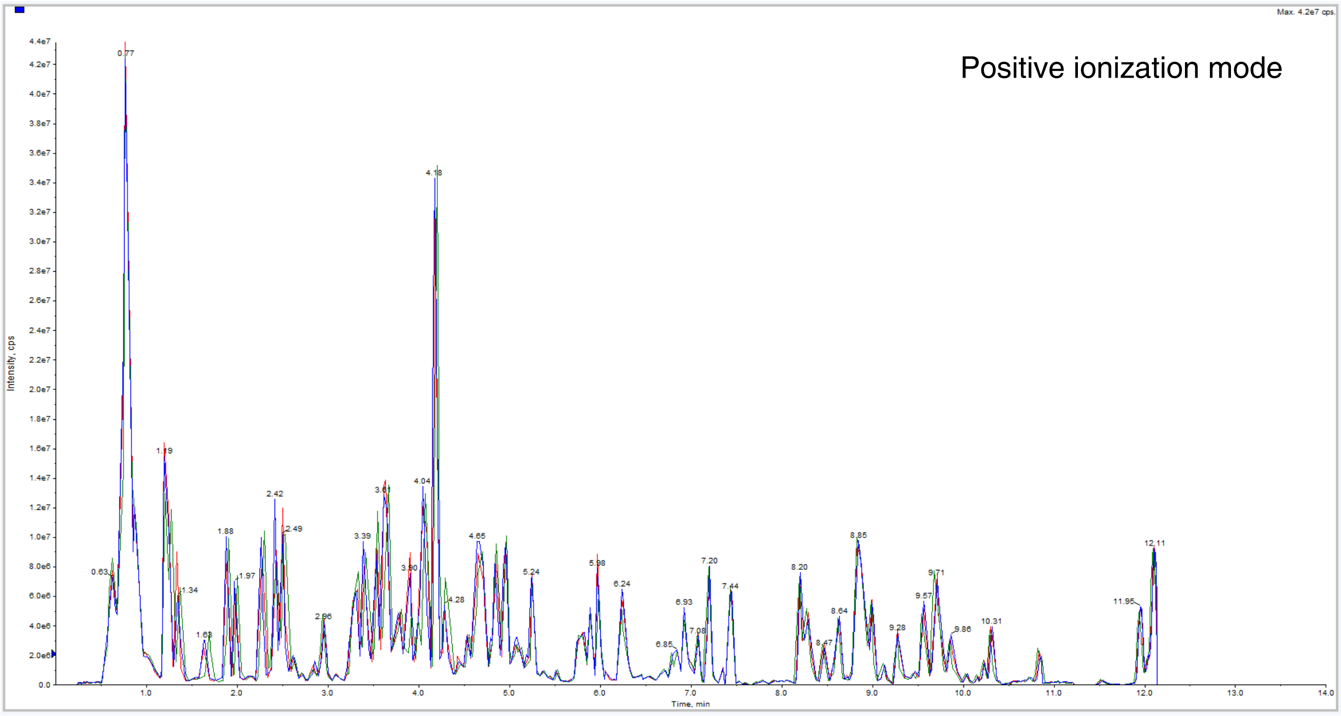

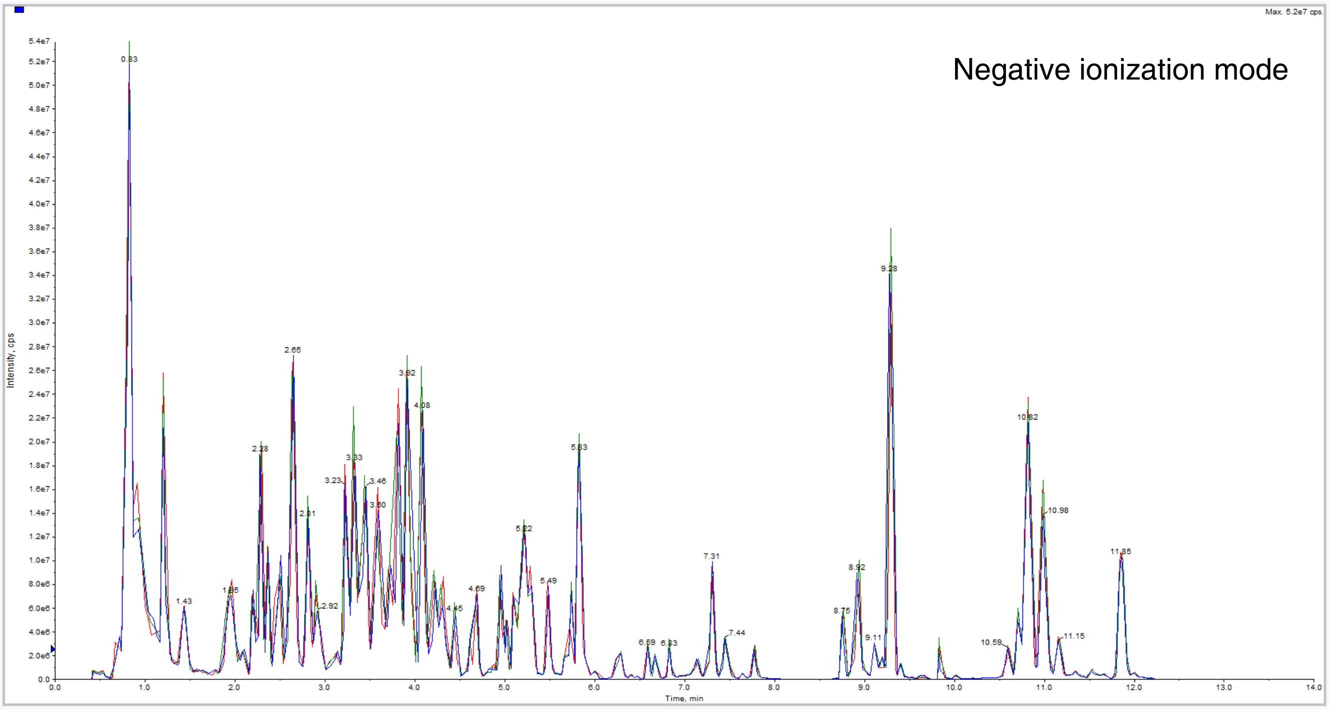

Fig. S5 Overlapping TIC of the QC mixtures in positive and negative ionization modes in the widely targeted metabolome.

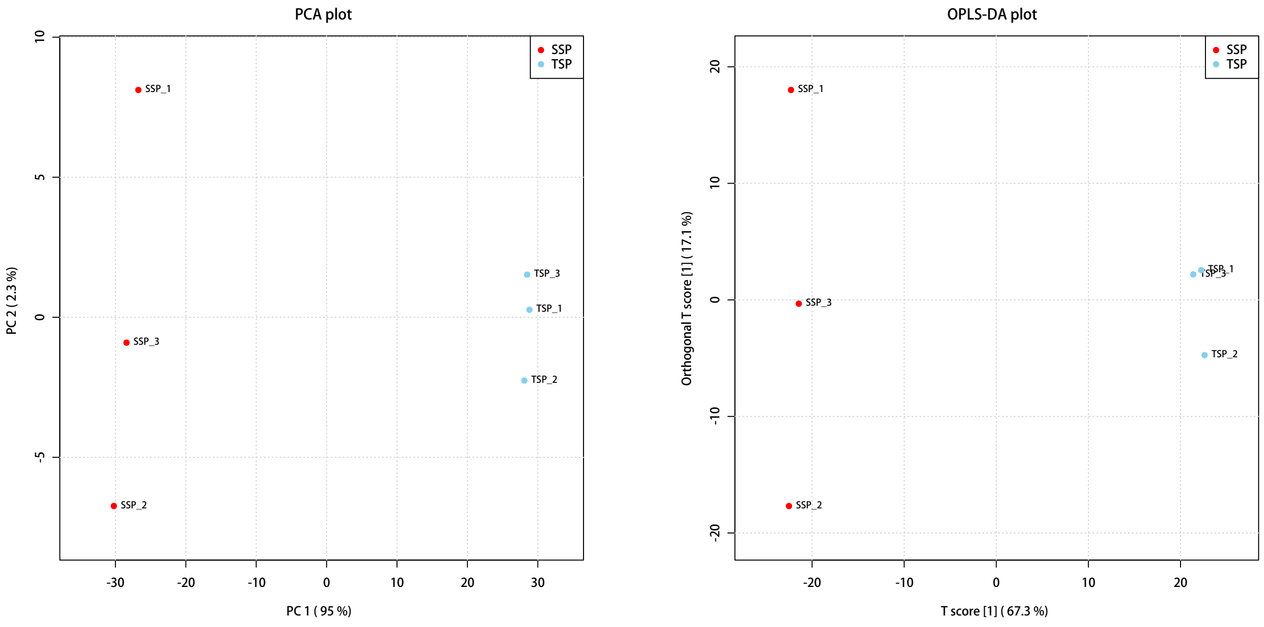

Fig. S6 PCA and OPLS-DA plots of the widely targeted metabolome data.

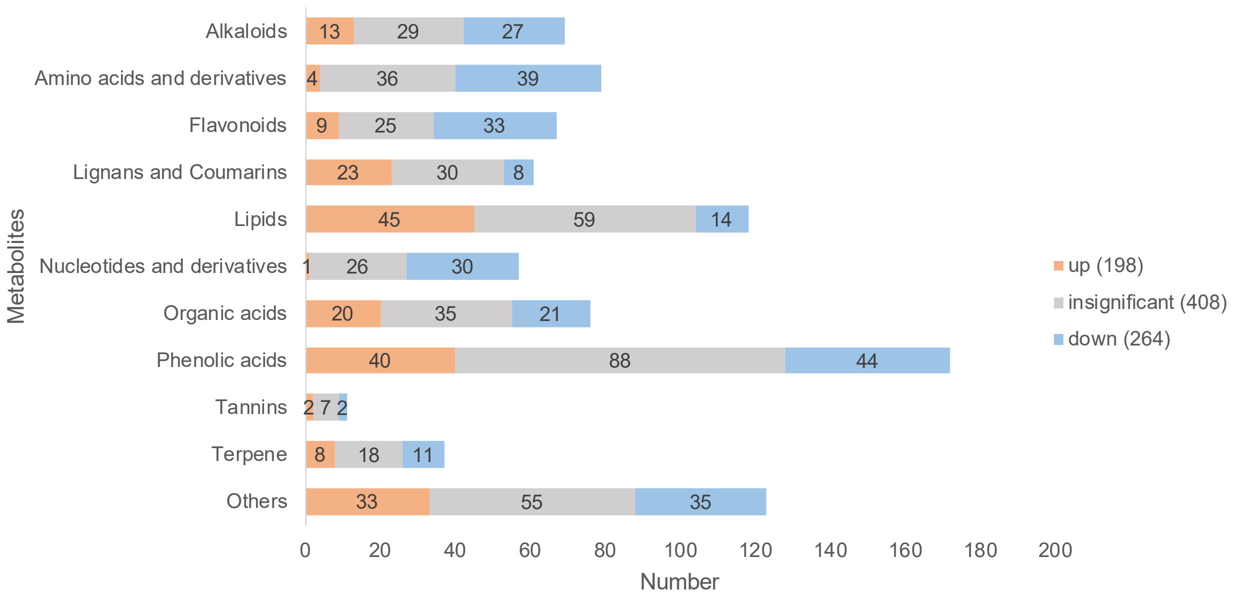

Fig. S7 Differentially accumulated metabolites in the widely targeted metabolome.

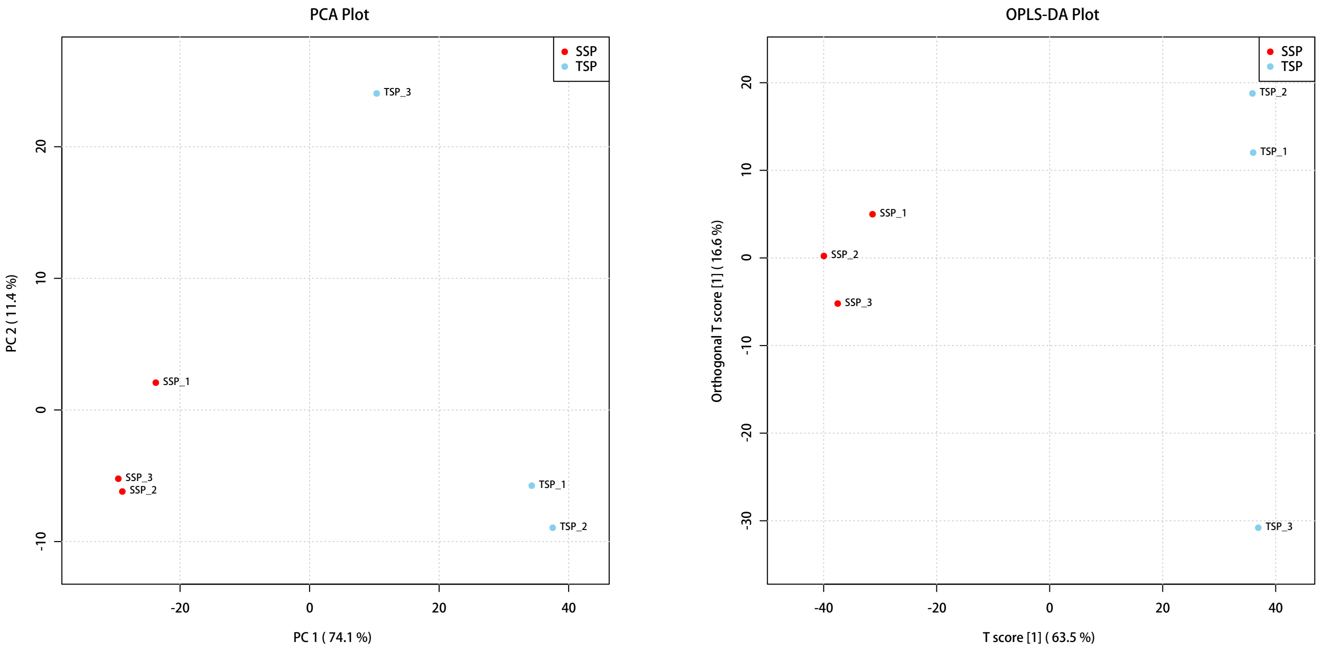

Fig. S8 PCA and OPLS-DA plots of the transcriptome data.

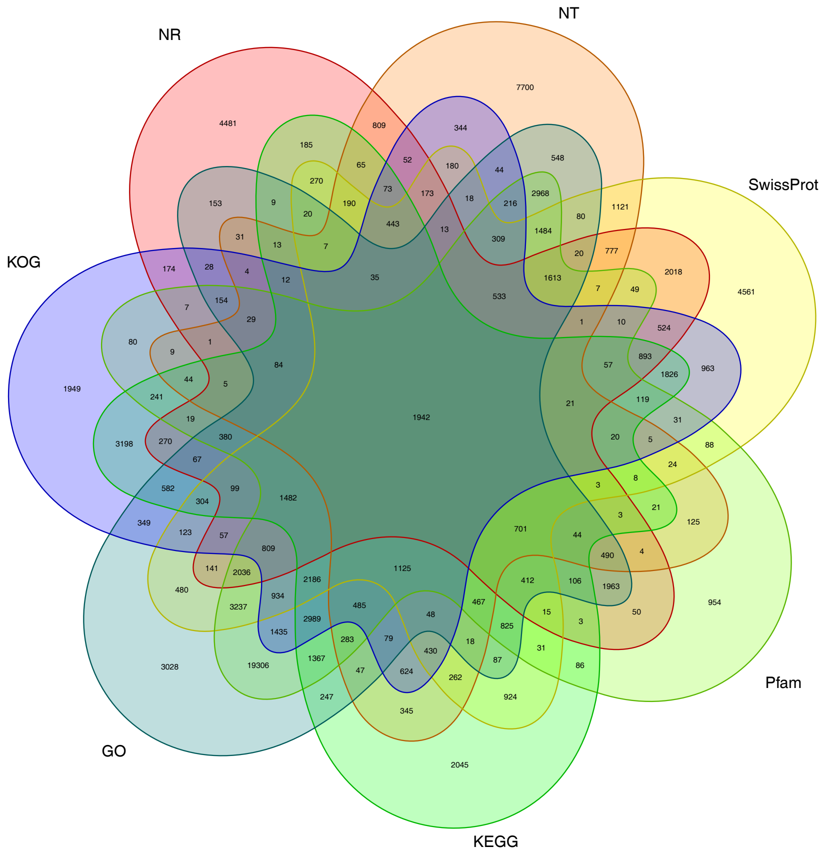

Fig. S9 Venn plot of the genes annotated in the NR, NT, Pfam, SwissProt, KEGG, GO, and KOG databases.

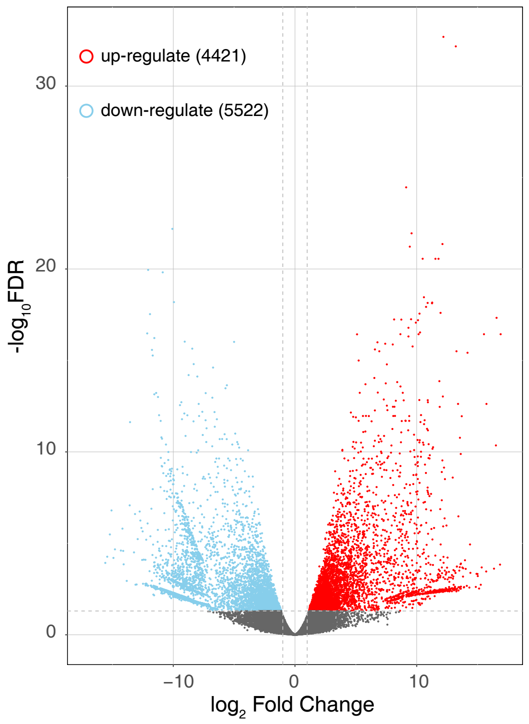

Fig. S10 Volcano plot of differentially expressed genes (DEGs) in SSP and TSP.

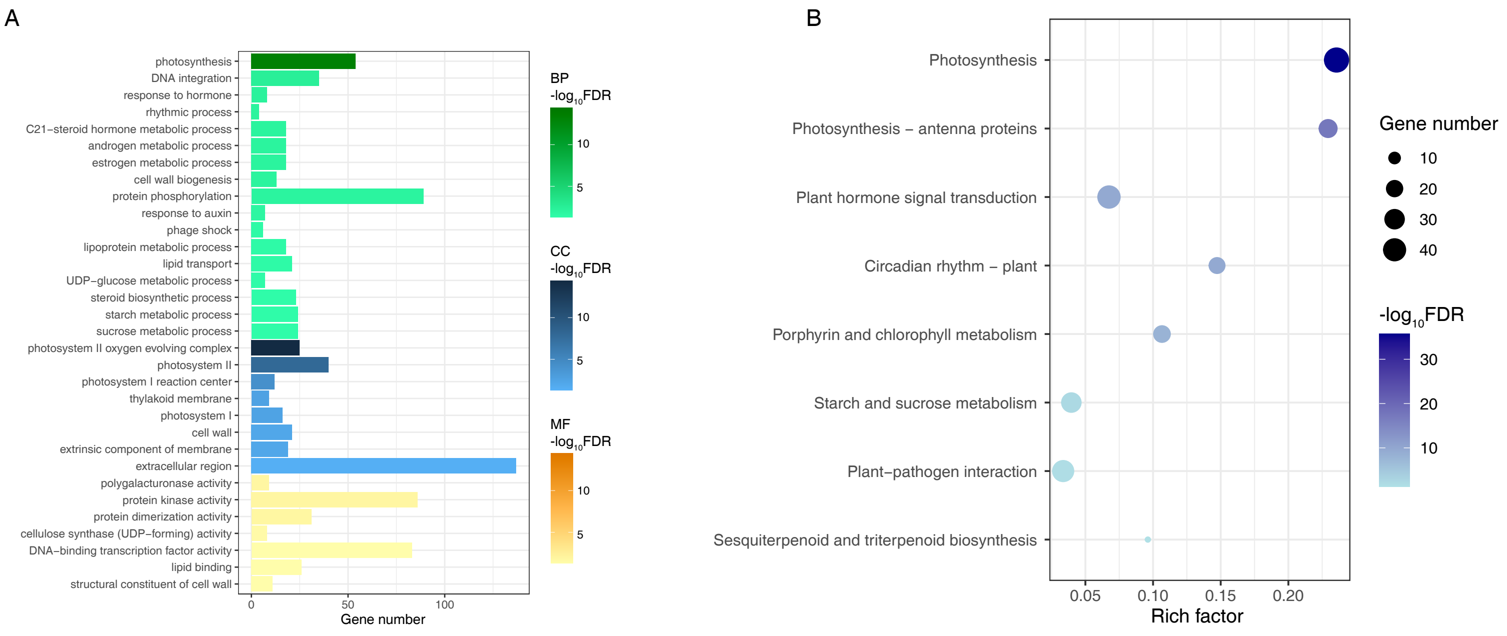

Fig. S11 Enrichment analyses of downregulated DEGs. (A) GO enrichment analysis of upregulated DEGs in TSP. (B) KEGG enrichment analysis of upregulated DEGs in TSP.

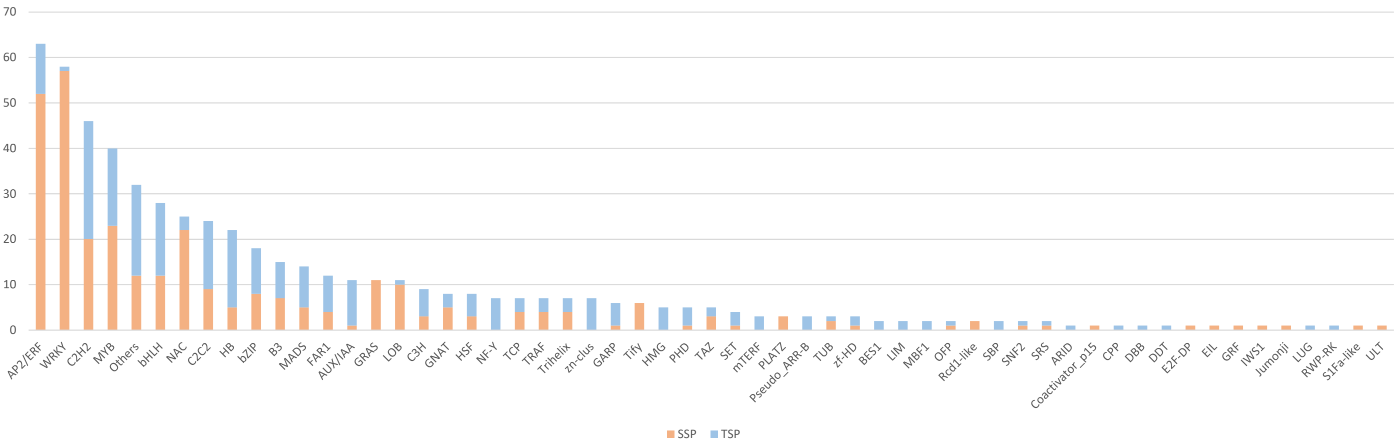

Fig. S12 Differentially expressed transcription factors.

Table S1-S3 are provided as Excel files.

Table S4 Detailed information on the sequencing data.

Sample	Raw reads	Clean reads	Clean bases (G)	Error rate	Q20	Q30	GC content
MSP_1	54438044	52993900	7.95	3.00%	97.21%	92.16%	43.03%
MSP_2	67521420	66485950	9.97	3.00%	97.93%	93.78%	43.10%
MSP_3	42488156	42033684	6.31	2.00%	98.20%	94.48%	43.35%
YSP_1	50829146	49878280	7.48	3.00%	97.84%	93.67%	42.11%
YSP_2	45308208	44112694	6.62	3.00%	97.96%	93.98%	44.37%
YSP_3	428,45,698	41793418	6.27	3.00%	97.94%	93.92%	43.11%

Table S5 Detailed information on the assembly data.

Items	Transcripts
Minimum length (nt)	301
Mean length (nt)	1,023
Median length (nt)	635
Maximum length (nt)	21,148
N50 (nt)	1,532
N90 (nt)	426

Table S6 Potential differentially expressed sesquiterpene synthases.

Gene_id	Description	log2FC	P-value	FDR
Cluster-153981.7418	premnaspirodiene oxygenase	9.3234	1.0600E-20	1.6428E-17
Cluster-153981.7009	premnaspirodiene oxygenase	10.2767	1.9629E-20	2.9372E-17
Cluster-153981.6955	premnaspirodiene oxygenase	10.1570	2.8814E-20	3.6776E-17
Cluster-153981.6173	premnaspirodiene oxygenase	8.6624	2.8597E-20	3.6776E-17
Cluster-153981.85372	vetispiradiene synthase	13.2685	3.4818E-19	3.1478E-16
Cluster-153981.4784	vetispiradiene synthase	13.7024	2.7084E-15	1.1193E-12
Cluster-153981.6892	Trans-alpha-bergamotene synthase	8.0054	1.6195E-14	5.5776E-12
Cluster-153981.6616	premnaspirodiene oxygenase	5.5693	2.2415E-13	5.7218E-11
Cluster-153981.6242	(3S,6E)-nerolidol synthase	8.6070	5.3308E-13	1.2240E-10
Cluster-153981.7010	premnaspirodiene oxygenase	11.0758	6.6957E-13	1.5213E-10
Cluster-153981.85371	vetispiradiene synthase	10.7771	1.5678E-09	1.3689E-07
Cluster-153981.6243	(3S,6E)-nerolidol synthase	7.4150	2.2155E-07	9.2267E-06
Cluster-153981.4283	premnaspirodiene oxygenase	9.9421	2.6710E-07	1.0871E-05
Cluster-153981.6954	premnaspirodiene oxygenase	6.4878	3.6114E-07	1.4081E-05
Cluster-173622.2	premnaspirodiene oxygenase	6.4587	4.7879E-07	1.7989E-05
Cluster-173622.3	premnaspirodiene oxygenase	9.9101	1.5433E-05	3.1649E-04
Cluster-154974.0	alpha-farnesene synthase	5.2543	1.4697E-04	1.9303E-03
Cluster-153981.7122	premnaspirodiene oxygenase	5.7065	2.8313E-04	3.2340E-03
Cluster-173622.0	premnaspirodiene oxygenase	10.4274	4.0227E-04	4.1682E-03
Cluster-153981.28815	Germacrene synthase	-5.1130	8.5535E-07	2.9272E-05
Cluster-153981.30969	NAD+-dependent farnesol dehydrogenase	-2.9166	6.9550E-06	1.6358E-04
Cluster-153981.54993	costunolide synthase	-2.8824	4.7823E-04	4.7391E-03
Cluster-153981.44878	premnaspirodiene oxygenase	6.3568	4.9589E-04	4.8620E-03
Cluster-153981.12195	beta-caryophyllene synthase	-4.6799	1.4353E-03	1.0665E-02
Cluster-153981.20934	costunolide synthase	-3.4352	2.0732E-03	1.3888E-02
Cluster-153981.20308	beta-caryophyllene synthase	-4.2893	5.6241E-03	2.8508E-02
Cluster-153981.59873	5-epiaristolochene synthase	-2.7712	7.0453E-03	3.3970E-02
Cluster-153981.84157	Trans-alpha-bergamotene synthase	6.3313	7.0507E-03	3.3985E-02
Cluster-153981.28816	Germacrene synthase	-4.4575	1.0201E-02	4.5610E-02

Table S7 Differentially expressed *MAPKs*.

Gene_id	Description	log2FC	P-value	FDR
Cluster-153981.20612	MAPK9(2)	6.824301	2.62E-12	4.95E-10
Cluster-153981.13887	MAPK9(1)	3.828166	3.11E-07	1.24E-05
Cluster-153981.8988	MAPK(2)	4.778011	3.33E-05	0.00059
Cluster-153981.6627	MAPKKK1(2)	2.452361	0.000476	0.004723
Cluster-153981.46805	MAPK19	2.007098	0.000667	0.006059
Cluster-153981.35336	MAPK5	2.07255	0.000789	0.006867
Cluster-153981.59382	MAPK1	2.440717	0.001339	0.010164
Cluster-153981.58872	MAPK2(1)	2.592854	0.001493	0.010956
Cluster-153981.8935	MAPK2(2)	2.488372	0.002015	0.013621
Cluster-153981.54398	MAPKKK3	1.404263	0.004132	0.022248
Cluster-153981.21228	MAPK(1)	-1.6474	0.006273	0.03103
Cluster-195221.0	MAPK(3)	-3.21441	0.010647	0.047163
Cluster-153981.51424	MAPKKK1(1)	-1.90023	0.011072	0.048671

Table S8 Differentially expressed *PRs*.

Gene_id	Description	logFC	FDR	P_value
Cluster-153981.4207	PR3(2)	10.10775	6.3E-18	3.77E-21
Cluster-153981.3569	PR3(1)	5.948701	7.98E-09	6.1E-11
Cluster-153981.15313	PR10	6.706498	8.37E-09	6.46E-11
Cluster-153981.3747	PR6	7.390667	1.4E-07	1.61E-09
Cluster-153981.63259	PR9(3)	6.409088	3.76E-07	5.06E-09
Cluster-153981.8121	PR9(2)	5.111448	2.33E-06	4.35E-08
Cluster-153981.4209	PR8	5.151616	3.31E-05	9.96E-07
Cluster-153981.11651	PR5(3)	5.184597	5.89E-05	2.03E-06
Cluster-143239.0	PR5(2)	10.00635	0.000119	4.66E-06
Cluster-163904.0	PR5(1)	5.510944	0.000274	1.3E-05
Cluster-153981.9340	PR9(1)	4.331035	0.000776	4.69E-05
Cluster-153981.3291	PR17	3.673268	0.005559	0.000594
Cluster-153981.39173	PR1	-4.01488	0.01127	0.001552
Cluster-153560.0	PR3(3)	5.739396	0.031917	0.006507

Table S9 Primers for qRT-PCR.

Gene name		Primers（5'-3'）
TBP	F	AATTGGCTGCTCGGAAGTATG
	R	CGTAACTTGAAAAGGCACCATG
PAL	F	ACGGAGTTATGTTGTGGTTGAGG
	R	AAGAGCAGCCATGCTCGTTC
CSE	F	TTGACAGTTCACGGGACATCA
	R	CCATTACCCTTTGGACCATACC
C4H	F	ACTAATGGCGGAAAACGGAG
	R	GGCCTTTAAGGACGGCTGA
ACAT	F	CTAAGATCAAAGCAGCAGCACC
	R	TGCAGGTTGAAGTATCTGGAGG
MVK	F	AGTGAGTTGGCTTCCATCATCC
	R	TCCACCACCAGCTCCTGTTAA
MVD	F	GCCCGCATCTTGTAATATCGT
	R	ATAGAAACACCGCTGCCCTC
HMGR	F	GACATTTTGGATGAGTTTGAGGAG
	R	GTTTGGATTTAGGTTTGGGAGAAG
SQS	F	ATTCACGACGACTCACCATCAC
	R	GGGACTCTGGAACCTTAAACGA
DDS	F	CAAGACAATAATCAGTGGAGGAGTG
	R	GAAGAAGTAACCTATGAAACAGCGAC
GID1B	F	CCTGATGGAAACAAACCAGTTG
	R	GGTGATACGAGGTCCCGAGTAC
PLY8	F	AGACTTAGAAACTACTCTTCCACCAT
	R	TCCCTTCAGGCACATCCACT
LHCA2	F	GGATCAGCAAGGTGAGCAAAG
	R	AGTGAAGGAATTGAGGACAAAGG
LHCA4	F	CACTACGTCGAGATCAGAAGGTG
	R	CAAATCCCAGGAATGCCAAC
PSBR	F	AGAAGATCAAGACCGACAAGCC
	R	CTGCCAGAAGCATCCAGACC
PETC	F	AGATGCCAGGCTACCAGTGTTC
	R	TGCCAGCAGTGGGAAGTGA
GAUT	F	ACTGGTGAATCTGGAAATGTTGC
	R	ATCTAATGCCCTGCGTGCC
